# The Third National Telemedicine & Telehealth Service Provider Showcase Conference: Advancing Telehealth Partnerships

**DOI:** 10.1089/tmj.2018.0096

**Published:** 2019-04-10

**Authors:** Dale C. Alverson, Elizabeth A. Krupinski, Kristine A. Erps, Nancy S. Rowe, Ronald S. Weinstein

**Affiliations:** ^1^ New Mexico Telehealth Alliance, Albuquerque, New Mexico.; ^2^ Emory University, Atlanta, Georgia.; ^3^ Arizona Telemedicine Program, The University of Arizona, Tucson, Arizona.; ^4^ Department of Pathology, The University of Arizona, Tucson, Arizona.

**Keywords:** *telemedicine*, *telehealth*, *education*, *commercial telemedicine*

## Abstract

***Introduction:*** *As telemedicine and telehealth services are experiencing increasing rates of adoption, industry leaders and healthcare service providers are becoming increasingly focused on human resource issues encountered in the delivery of a broad range of telehealth services. To create a forum for the discussion of many interrelated elements of telehealth service industry, a national conference entitled “Telemedicine & Telehealth Service Provider Showcase” (SPS) Conference was established in 2014, and repeated in 2016 and 2017, in Arizona. These SPS Conferences include thought leaders, telehealth service providers, government administrators, and academicians from leading programs addressing service provider workforce issues.*

***Methods:*** *This report summarizes the content of SPS 2017 conference, held in Phoenix, AZ, October 2–3, 2017. The topics covered at SPS 2017 include using telehealth services as a strategic asset; development of appropriate effective partnerships; direct-to-consumer initiatives; important reimbursement, legislative, and regulatory issues (i.e., Centers for Medicare & Medicaid Services [CMS] approaches, financial models, and return on investment [ROI]); marketing; evaluation and applied metrics; remote monitoring and sensors; integration with electronic health records; and overall lessons learned.*

***Results:*** *The content of SPS 2017 is summarized in the body of this report. The SPS 2017 program evaluators included attendees, speakers, and exhibitors. The knowledge attendees gained at SPS 2017 was characterized, by all three groups, as forward-looking and practical.*

***Conclusion:*** *SPS 2017 succeeded in identifying, and focusing on, solutions for issues, challenges, and barriers impacting the rapidly expanding telehealth service segment of the healthcare industry. The growing interest in this annual SPS Conference series apparently reflects, in part, the program committee's successes in identifying practical issues and their potential solutions.*

## Introduction

Telemedicine and telehealth (hereafter telehealth) are increasingly being adopted and integrated into our community, regional, national, and international healthcare systems. As the telecommunications infrastructure improves, telecommunications systems are becoming less expensive and easier to use, including incorporating users' own devices such as mobile phones and tablets along with cloud-based solutions.^1–5^ Telehealth offers tools to address the “quadruple aim” of improving the patient experience, improving population outcomes, decreasing cost without sacrificing quality, and improving provider experiences.^6,7^ Increasingly, data are demonstrating positive impacts of telehealth in these domains, including improved patient care and health outcomes in addition to cost savings and cost avoidance.

Recognizing the emerging phenomenon of vendors/industry and healthcare systems providing healthcare services leveraging telehealth, as opposed to just providing equipment and software, the Telemedicine & Telehealth Service Provider Showcase (SPS) Conference was developed. It is the brainchild of Ronald S. Weinstein, MD, Director of the Arizona Telemedicine Program (ATP) and Co-Director of the Southwest Telehealth Resource Center (SWTRC). The SPS Conference series is a companion program for the ATP's successful on-line Service Provider Directory (SPD), one of the telehealth industries' largest listing of telehealth service companies, available on the internet.^8^ SPS is supported and co-chaired by Elizabeth A. Krupinski, PhD and Dale C. Alverson, MD. The conference was designed to explore and demonstrate successful service provider approaches, provide examples of successful partnerships, assess ongoing challenges, and propose solutions.

The conference exhibition hall showcases a broad spectrum of telemedicine service providers. It provides opportunity for one-on-one networking.

**Fig. 1.**
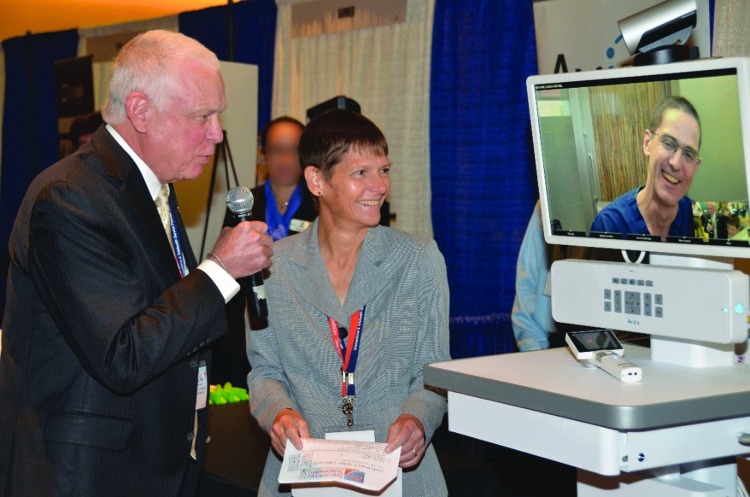
Co-Chairs of SPS, Drs. Elizabeth A. Krupinski and Dale C. Alverson (left), interviewing a vendor (right, video monitor, Alan Pitt, MD, Chief Medical Officer of Avizia) in the Expo Hall, broadcasting their string of focused “What's New?” interviews back to the lecture hall during the SPS “Lightning Rounds.” These proved to be popular, and informative, events and an efficient way to introduce SPS Conference attendees to the 40 exhibitors at SPS 2017. SPS, Telemedicine & Telehealth Service Provider Showcase. Used by permission.

A popular feature of SPS Conferences, designed to help attendees navigate the Exhibition Hall is the “Lightning Rounds.” These highlight exhibitors one-at-a-time, allowing them to give a brief description of their products and services to the entire audience. These live video interviews are transmitted from the Exhibition Hall to the audience in the lecture theater, so the audience can preview the exhibitors up front and prioritize which exhibitors to visit and interact with during breaks. Attendees see, and hear firsthand, what these “best of breed” telehealth companies have to offer (*Fig. 1*).

## Conference Summary: Highlights SPS 2017

*Table 1* displays the overall SPS 2017 Program.

**Table 1.** SPS 2017 Conference Schedule at a Glance

MONDAY, October 2, 2017	
8:00–8:10	Welcome • Ronald S. Weinstein, MD, Arizona Telemedicine Program, Dale C. Alverson, MD, University of New Mexico & Elizabeth A. Krupinski, PhD, Southwest Telehealth Resource Center
8:10–8:40	Keynote Address—Telehealth as a Strategic Asset: Are You Ready? • Alan C. Roga, MD, Teladoc
Module 1/Telehealth Winners: Strategies that Work Section Moderator: Ronald S. Weinstein, MD, Arizona Telemedicine Program	
8:40–9:10	Saving a Rural Hospital through Telemedicine • James J. Dickson, MBA, Copper Queen Community Hospital
9:10–9:35	Meet the Service Providers Lightning Rounds Dale Alverson & Elizabeth Krupinski with exhibitors
9:35–10:05	Networking Break—Expo Hall *Sponsored by Avizia*
Section Moderator: Alexis Gilroy, JD, Jones Day	
10:05–11:05	Panel—Finding and Vetting Your Perfect Telehealth Partner • Alexis S. Gilroy, JD, Jones Day • Elizabeth A. Krupinski, PhD, Southwest Telehealth Resource Center • Sarah N. Pletcher, MD, MHCDS, Geisel School of Medicine at Dartmouth
11:05–11:35	Embracing Direct-to-Consumer Telemedicine Beyond On-Demand Brian Wayling, MBA, Intermountain Healthcare
11:35–12:05	Championing Telehealth Legislation in Your State Kofi Jones, KJ Health Matters
12:05–1:20	Lunch—Expo Hall
Module 2/Legal and Regulatory Considerations— Section Moderator: Sarah N. Pletcher, MD, MHCDS, Geisel School of Medicine at Dartmouth	
1:20–2:35	Panel—A Deep Dive into Advanced Telehealth Legal and Regulatory Issues • Maureen Cahill, MSN, APN-CNS, AOCNS, National Council of State Boards of Nursing • Michael Grafton, US Drug Enforcement Agency/Phoenix Field Office • Nathaniel Lacktman, JD, Foley & Lardner, LLP
2:35–3:00	Meet the Service Providers Lightning Rounds • Dale Alverson & Elizabeth Krupinski with exhibitors
3:00–3:30	• Networking Break—Expo Hall *Sponsored by the Good Samaritan Society and Trapollo*
Section Moderator: Nathaniel Lacktman, JD, Foley & Lardner, LLP	
3:30–4:00	Understanding Medicare Fine Print • Tracy Schutt, Noridian Healthcare Solutions • Tana Williams, Noridian Healthcare Solutions
4:00–5:00	Panel: Coverage and Payment: Negotiating Win-Wins • Claire E. Castles, JD, LLM, Jones Day • Cynthia Hatcher, WellCare Health Plans • Nathaniel Lacktman, JD, Foley & Lardner, LLP
5:00–6:30	Networking Reception—Expo Hall *Sponsored by GlobalMed*
TUESDAY, October 3, 2017	
7:30–8:30	Poster Sessions and Breakfast—Lecture Hall
Module 3/Making Your Partnership Work Smoothly Section Moderator: B. Tilman Jolly, MD, Specialists On Call	
8:30–9:30	Panel—Show Me the Data! Outcomes and Savings from Telehealth Partnerships • Jason C. Goldwater, MA, MPA, National Quality Forum • Judd Hollander, MD, Thomas Jefferson University • Ryan McCool, MD, White River Junction Veterans Affairs Medical Center & Geisel School of Medicine at Dartmouth
9:30–10:00	Meet the Service Providers Lightning Rounds • Dale Alverson & Elizabeth Krupinski with exhibitors
10:00–10:30	Networking Break—Expo Hall *Sponsored by the Southwest Telehealth Resource Center*
Section Moderator: Amy L. Waer, MD, Arizona Telemedicine Program	
10:30–11:00	Successfully Marketing a Telehealth Partnership • Tammy Hatting, MPA, Avera eCARE
11:00–12:00	Panel—Integrating a Telehealth Partner into Your Organization: Best Practices and Lessons Learned • Robert Bernstein, MD, MPH, Carena • Chong Jacobs, RN, MSN, Banner Telehealth • Gigi Sorenson, RN, MSN, GlobalMed
12:00–1:15	Lunch—Expo Hall
Module 4/Navigating the Rapids of Changing Technology Section Moderator: Dale Alverson, MD, University of New Mexico	
1:15–1:45	Moving Away from “Platform Agnostic” • Bart M. Demaerschalk, MD, MSc, FRCP(C), Mayo Clinic Center for Connected Care
1:45–2:15	Case Study: On-Demand Digital Health Services • Shauna M. Coyne, New York-Presbyterian Hospital
2:15–2:45	Networking Break—Expo Hall
Section Moderator: Gigi Sorenson, RN, MSN, GlobalMed	
2:45–3:15	Integrating Telehealth into Your EHR • Andy Penn, Cerner Corporation
3:15–3:45	Wearable Sensors and Diabetic Foot Remission • David G. Armstrong, DPM, MD, PhD, Southwestern Academic Limb Salvage Alliance (SALSA), Keck School of Medicine at University of Southern California
3:45–3:55	Closing

EHR, electronic health record.

### Keynote: Telehealth As a Strategic Asset: Are You Ready?

The keynote presentation by Alan C. Roga, MD, addressed, head-on, the topic of “Telehealth as a Strategic Asset: Are You Ready?” Roga leads s successful direct-to-consumer (DTC) service known as “Teladoc^R^.” DTC's segment of the telehealth industry represents, in part, a response to consumer demand and desire for greater convenience in accessing providers through on-line systems. These programs respond to that need and address issues related to preserving standards of care, and integrating a new category of healthcare providers with the patient's primary care providers and medical home. Eventually, DTC services may become critical to the operations of many, if not most healthcare provider systems. Market forces may drive the increasing adaption of DTC services by integrated healthcare systems.

## Module 1: Telehealth Winners: Strategies that Work

### Saving a Rural Hospital Through Telemedicine

The first speaker in the “Telehealth Winners: Strategies that Work” module described a rural healthcare system and hospital that was “saved” through telehealth. As presented by James Dickson, CEO of Copper Queen Hospital (Bisbee, AZ), his rural hospital in Southeastern Arizona established a robust set of telemedicine services that successfully address the needs of the community and surrounding area. Their model demonstrates how a rural hospital can become a hub for telemedicine services, providing more comprehensive, evidence-based care to its patients, better support its providers, improve its own competitive position in the region, and reduce overall costs. Dickson is a seasoned innovator with extensive prior experience as an administrator in a large urban medical center.

“Telemedicine counterbalances disparities between urban and rural medical environments, results in better disease management, improves potential to practice evidence-based standards, and lowers cost on several levels.” According to Dickson several important “Lessons Learned” were as follows:

(1) Remember telemedicine is for the patient's benefit, not for profit. It is about the expansion of services that raise your profile as a healthcare facility.
(2) Develop protocols for which patients fit into the telemedicine program and can remain locally, or for those who should be transferred immediately.
(3) Remote physicians (the teleconsultants) should not expect to make a profit on the consult. They will make it on the referrals.
(4) Test your computer equipment regularly. The time for failure is not when the telephysician is in a consult.
(5) Train the remote telephysician and make it as easy as possible to perform the consult.
(6) Send documentation in advance whenever possible so that consult time is not wasted on the delivery of patient information.
(7) Talk to legislature and insurance companies to demonstrate the financial benefits to the insurance companies and for the patient not being transported to an urban hospital.
(8) Telemedicine requires commitment on the part of the remote provider for tele-emergency services when it may not always be convenient.
(9) Treating virtual patients like a regular visit will save the provider time. Stacking queued “virtual” patients who then are “no shows” wastes the provider's time.
(10) Adequate broadband, especially for stroke and CT images, is necessary. Telemedicine that does not require patient examination such as behavioral health or endocrinology can be performed using a wireless smart phone.

### Finding and Vetting Your Perfect Telehealth Partner

The first panel addressed the challenges of “Finding and Vetting Your Perfect Telehealth Partner” to form successful programs. Reasons and benefits for partnerships were discussed. Issues discussed further related to the legal implications of developing partnerships:

(1) doing due diligence to comply with legal and regulatory requirements and addressing risk management and malpractice issues;
(2) developing the appropriate contracts and defining joint obligations and responsibilities, and
(3) integrating telehealth into overall operations.

Suggested steps in approaching partnerships:

(1) doing what is best for your enterprise to best fulfill needs and meet provider and patient expectations;
(2) approach service providers as you would any other business partner–healthcare is a business;
(3) prepare and review contracts carefully;
(4) have quality assurance/quality control procedures in place with metrics;
(5) suggest a trial period before fully committing; and
(6) train, prepare, and again, integrate!

### Embracing DTC Telemedicine Beyond On-Demand

This session introduced the concept of how DTC telehealth can be more fully integrated into an existing healthcare delivery system, and was presented by Brian Wayling, Assistant VP, Telehealth Services, Intermountain Healthcare in Utah. Intermountain, a large integrated healthcare system, has experienced significant growth in its' spectrum of DTC initiatives, including “on-demand” patient consultations, remote patient monitoring, scheduled telemedicine visits, personal wellness, and preventive care interventions. Although Intermountain has faced challenges, they are experiencing cost savings that outweigh the costs of the programs and add value to their system, including integration of telehealth with their electronic health record (EHR).

### Championing Telehealth Legislation in Your State

This module was rounded out with a presentation on how best to develop and advocate for legislation that supports telehealth and how to engage the legislators. Finding legislative “champions” can facilitate development of high impact telehealth legislation at a state level.

## Module 2: Legal and Regulatory Considerations

### A Deep Dive Into Advanced Telehealth Legal and Regulatory Issues

The first session of the second module, “Legal and Regulatory Considerations,” had a panel exploring advanced telehealth legal and regulatory issues, including nonphysician providers in telehealth, telemedicine prescribing, and using telehealth to build a stronger medical program. Maureen Cahill, Senior Policy Advisor of the National Council of State Boards of Nursing (NCSBN), discussed the value of the nursing compact that facilitates nurse licensing among Compact states, in addition to education and certification regarding advanced practice registered nurses. She presented the nursing compact as being similar to having a driver's license: “state-based, nationally recognized, and locally enforced.” Criminal background checks are now required for all Compact states, with felony convictions a bar to a Compact license. Various provisions improve the operations of the Compact, and the NCSBN commits to fund ongoing operations of the Compact and assist states with grants for implementation expenses. Nursing will play an important role in the delivery of a wide spectrum of telehealth services within and between states.

Michael Grafton of U.S. Drug Enforcement Agency (DEA), Phoenix Field Office, discussed issues related to prescribing medication when using telemedicine. Telemedicine providers must be aware of the federal and state rules, regulations, and restrictions regarding providing prescriptions in conjunction with a telemedicine encounter.

Nathaniel Lacktman, JD, Partner, Foley and Lardner, LLP, of Tampa, FL, discussed the elements of building “destination medicine programs” that leverage the integration of telehealth. He pointed out that healthcare consumers will be looking for high quality, no wait, world class facilities, access to the latest technology, best doctors, and “concierge” level care over a spectrum of health services. Telehealth will provide the tools to make a healthcare system a destination for those health services. Several national and international legal and regulatory issues will require attention, but can lead to multistate and international initiatives to facilitate the development of becoming a true destination for healthcare.

### Understanding Medicare Fine Print and Other Payment and Coverage Approaches: Negotiating Win-Wins

Tracy Schutt and Tana Williams, Provider Outreach & Education representatives of Noridian Healthcare Solutions, of Fargo, ND, gave presentations that addressed understanding Medicare billing and payment specifics, other payment and coverage approaches, and Medicaid products. Best approaches to reimbursement and parity laws were discussed, along with specific examples including “the good, the bad and the ugly” as related to the business of telehealth and sustainability. Noridian Healthcare Solutions representatives provided details regarding programs related to Medicare and application of telehealth, including use of the telehealth service rendered via interactive audio and video telecommunications system and 95 modifiers for telehealth and applicable current procedural terminology codes. In addition, the originating site fee was discussed. They answered a series of questions related to telehealth and Medicare perspectives. Further issues related to auditing were flagged in addition to the Next Generation accountable care organization telehealth waivers.^9^ They also provided links to a variety of useful resources and educational tutorials.

Other contracting and reimbursement strategies were presented, including Centers for Medicare & Medicaid Services (CMS) demonstration programs and waivers, chronic care management, Medicaid managed care organization and Medicare Advantage programs, state coverage and payment, and other legislative initiatives.^10,11^ Several contract considerations were presented. Additionally, operational considerations were discussed to create a “win-win” outcome in negotiations with payers as organizations plan for a move into telehealth. Further presentations addressed the health plan perspective, including its value proposition, telehealth strategy, and approaches to innovation. Finally, policies relating to private payers were presented with consideration of stronger state parity laws and examples of stronger, more effective legislation, and less impactful legislative efforts related to reimbursement and coverage for telehealth services.

## Module 3: Making Your Partnerships Work Smoothly

### Show Me the Data! Outcomes and Savings from Telehealth Partnerships

Day 2 presentations started with the third module entitled, “Making Your Partnership Work Smoothly.” A panel on telehealth outcomes and savings data addressed the importance of evaluation and using appropriate metrics to demonstrate the value and return-on-investment (ROI) of a telehealth program, including evaluation approaches and the National Quality Forum (NQF) recent report as summarized by Jason Goldwater, Senior Director at NQF, Washington, DC, on appropriate, evidence-based telehealth metrics that can be used in evaluating the effects of telehealth in (1) access, (2) experience, (3) cost, and (4) effectiveness. These domains were further separated into sub-domains to provide a framework for applying evidence-based metrics in telehealth.^11^

(1) Access to Care:
  - access for patients or families (availability, affordability, accommodation, accessibility, appropriateness)
  - access for care team (provider adequacy)
  - access to information (medical records, pharmacy services, diagnostic tests)
(2) Financial Impact/Cost to:
  - patient, family, and/or caregiver
  - the care team
  - the health system or payer
  - society
(3) Experience:
  - patient, family, and/or caregiver
  - care team member including clinical provider (including tele-presenter)
  - community
(4) Effectiveness:
  - system effectiveness
  - clinical effectiveness
  - operational effectiveness
  - technical effectiveness

These data from the full NQF report will be critical in demonstrating the impact of telehealth on outcomes and savings coming from telehealth partnerships that can influence adoption and support.^12^

Judd E. Hollander, MD, of Thomas Jefferson University, in Philadelphia, PA, discussed the importance of aligning telemedicine operations with quality and outcomes, using an evidence-based framework as designed and outlined by NQF.^12^ That framework and approach can demonstrate the value and ROI to the healthcare provider institution and its leadership, which leads to sustainability and scalability.

Society for Education and the Advancement of Research in Connected Health (SEARCH) was presented as a grass-roots organization developed through the South Central Telehealth Resource Center and a number of key players in telehealth research.^13^ This society provides a platform to support advancing connected health initiatives and improving connected healthcare; unbiased, impartial research; education/training in connected health research methods; interpretation and dissemination; advancing evidence-based connected health; and informing health policy leadership on connected health research and its importance.

Ryan R. McCool, MD, an otolaryngologist at the White River Junction VA Medical Center, in White River Junction, VT, gave specific examples of data that demonstrated value and ROI through the use of telemedicine. They found the following: (1) there are significant time and travel savings for patients in rural settings; (2) organization savings are harder to quantify and tend to be system specific; and (3) telemedicine can be effective for evaluating most patients, serves as a powerful triage tool, and avoids more than 20% of unnecessary specialty referrals.

### Successfully Marketing a Telehealth Partnership

A presentation by Tammy Hatting, MPA, Director of Innovation for Avera eCare, of South Dakota included successful marketing of telehealth partnerships with other companies, integration into retail environments, and mobile systems that improve access to healthcare services and promote healthier lifestyles. Her takeaways were as follows:

(1) it's not about a telehealth visit—it's creating a destination and remarketing opportunities;
(2) marketing collaboratively is critical to driving traffic to the clinic and building the partnership–it takes a team;
(3) you cannot treat this as a stand-alone clinic—focus on conversion ratio;
(4) this is truly a new front door to your medical group to draw in new patients; and
(5) the ROI on the clinic is hard to see, but the downstream revenue is in millions!

### Integrating a Telehealth Partner Into Your Organization: Best Practices and Lessons Learned

In this session, a panel addressed best practices and lessons learned regarding integrating a clinical teleservices partner into a healthcare system from a healthcare delivery systems perspective. Robert Bernstein, MD, MPH, Vice President of Clinical Affairs for Carena, based in Seattle, WA, presented Carena's approach to integrating telehealth with health systems:

(1) partner with health systems to implement and operate their own branded virtual clinics, primarily for on-demand virtual urgent care services;
(2) have patient-initiated visits using consumer devices to access care 24/7—a health-system-branded DTC service;
(3) provide software services, digital marketing, administrative support, and clinical expertise; and
(4) staff clinics with Carena medical group clinicians, local health system clinicians, or a hybrid model using both.

He emphasized the importance of clinical protocols that are evidence-based and adapted to telemedicine, and appropriate training along with integration into the EHR and overall workflow, documentation and communication, and managing the patient as an “activated consumer.”

Deborah H. Dahl, BSE, MBA, VP of Patient Care Innovation at Banner Health, in Phoenix, AZ, pointed out that “one size doesn't fit all” and telehealth needs to be customized to the healthcare facility, whether it is large, medium, small, or a critical access hospital. She further pointed out the need to identify key players: project manager, technology management members, nursing leaders, physician champions, staff champions, and educators. Banner's lessons learned included the folowing:

(1) involve all players early on,
(2) set clear expectations,
(3) include pretesting and a final check,
(4) integrate new leaders and employees,
(5) create a technical support plan, and
(6) develop a reporting system.

Gigi Sorenson, RN, MSN, Director of Clinical Integration at GlobalMed, noted the importance of considering the hub and spoke perspectives in a telehealth network, based on her recent experience at Northern Arizona Healthcare partnering with a clinical teleservice provider. Her lessons learned included the following:

(1) have no preconceived ideas—stereotypes are out the window—prepare to be amazed;
(2) the process is more complicated than expected;
(3) connectivity will get you most times; and
(4) you can never communicate enough or be too involved.

## Module 4: Navigating the Rapids of Changing Technology

### Moving Away from “Platform Agnostic”

The fourth and final module of the conference, “Navigating the Rapids of Changing Technology,” started off with a provocative presentation by Bart M. Demaerschalk, MD, MSc, of the Mayo Clinic related to moving away from a “Platform Agnostic” approach to provide more telehealth operational consistency, interoperability' and reliability of the telehealth technologies, infrastructure, and networks in a large healthcare system with multiple sites. He also addressed the problems with multiple products and high failure rates. Mayo's approach resulted in the following:

(1) optimized network connections, standardization of products across all acute care telemedicine services;
(2) deployment to nine emergency departments and labor and delivery units across Arizona and the Midwest, implementation of a 24 × 7 support model;
(3) standardized contracts and service level agreement language, administrative operations convergence; and
(4) clinical service activation centralization. This led to a significant reduction in technical issues, dropping from 27% to 3%.

In conclusion, Mayo's approach of progressing from “service line” to “enterprise model” required convergence and centralization of technology, people, operations, governance, and evaluative strategies.

### Case Study: On-Demand Digital Health Services

Shauna M. Coyne, BSc. Director, IT Innovation, New York Presbyterian Hospital, in New York City, presented a model for a regional healthcare system that improved access and efficiency. Their approach included (1) access to a second opinion, digital emergency, and urgent care; (2) digital scheduled visits; and (3) digital physician-to-physician consults regarding patient cases. It led to fast-tracking low-acuity patients, reduced wait times, and virtual medical screening exams (average seven patients per hour). Urgent care visit times were 8–10 min, on average. Scheduled visits cover a broad spectrum of specialty healthcare services, and specialty consults between physicians reduced transfers, reduced readmissions, and leveraged existing specialists as New York Presbyterian expands its enterprise. Their approach involved 80% people, 15% process, and 5% technology.

### Integrating Telehealth Into Your Electronic Health Record

Andrew Penn, FCCA, MBA, Senior Director of the Strategic Business Unit of Cerner Corporation, of Kansas City, MO, addressed the importance of integrating telehealth with one's EHR and health information exchange. He emphasized the need for access to a patient's health information during a telemedicine visit similar to what would be needed during an in-person visit, and providing an integrated platform for documentation accessible by others involved in the patient's care and part of the medical home. Methods for accomplishing this integration were presented.

### Wearable Sensors and Diabetic Foot Readmission

The final presentation of the SPS Conference, on the successful use of wearable sensors and remote monitoring, was discussed by David C. Armstrong, DPM, MD, PhD, Professor of Surgery and Director of the Southwestern Academic Limb Salvage Alliance (SALSA), of Los Angeles, CA. He described a significant decrease in readmissions for diabetic foot disease by using sensors to detect sensory-neuro deficits and circulatory problems in a diabetic patient's feet early and help avoid foot amputations or the need for readmission.

## Conference Evaluations

SPS provided a practical perspective for partnering to integrate telehealth services into a wide spectrum of healthcare delivery systems, including: relevant models; important related regulatory and policy issues that should be addressed: and real-life experiences and practical advice. In the 17 question conference evaluations, attendees rated their experience “high” in meeting their expectations as they develop telehealth programs in their own systems, with an average rating of 4.69 on a 1–5 Likert Scale with 5, being the highest rating. Attendees stated that the conference was “relevant,” “practical,” and “comprehensive,” in the free-text section of the evaluation form.

## References

[1] Gagnon MP, Duplantie J, Fortin JP, et al. Implementing telehealth to support medical practice in rural/remote regions: What are the conditions for success? Implement Sci 2006**;** 1:18 16930484 10.1186/1748-5908-1-18 PMC1560157

[2] Doarn CR, Pruitt S, Jacobs J, et al. Federal efforts to define and advanced telehealth—A work in progress. Telemed J E Health 2014**;** 20:409–418 24502793 10.1089/tmj.2013.0336 PMC4011485

[3] Institute of Medicine (IOM). The role of telehealth in an evolving healthcare environment: Workshop summary. Washington, DC: National Academies Press, 2012 24901186

[4] Bashshur RL, Shannon GW, Smith BR, et al. The empirical foundations of telemedicine interventions for chronic disease management. Telemed J E Health 2014**;** 20:769–800 24968105 10.1089/tmj.2014.9981 PMC4148063

[5] Hollander JE, Carr BG Transforming care delivery through telemedicine. In: Wiler JL, Pines JM, Ward MJ, eds. Value and quality in acute and emergency care. New York: Cambridge University Press 2017:131–138

[6] Berwick DM, Nolan TW, Whittington J The triple aim: Care, health, and cost. *Health Aff* (Millwood) 2008**;** 27:759–769 18474969 10.1377/hlthaff.27.3.759

[7] Bodenheimer T, Sinsky C From triple to quadruple aim: Care of the patient requires care of the provider. Ann Fam Med 2014**;** 12:573–576 25384822 10.1370/afm.1713 PMC4226781

[8] Arizona Telemedicine Program's Service Provider Directory. Available at https://telemedicine.arizona.edu/servicedirectory (last accessed 42, 2018)

[9] Center for Medicare & Medicaid Services–Accountable Care Organizaions (ACOs). Available at https://innovation.cms.gov/initiatives/ACO (last accessed 327, 2018)

[10] Centers for Medicare & Medicaid Services (CMS). MACRA delivery system reform, Medicare payment reform: What's the quality payment program? Available at https://www.cms.gov/Medicare/Quality-Initiatives-PatientAssessment-Instruments/Value-Based-Programs/MACRAMIPS-and-APMs/MACRA-MIPS-and-APMs.html (last accessed 12017)

[11] CMS. Next generation ACO model website. Available at https://innovation.cms.gov/initiatives/Next-Generation-ACO-Model (last accessed 42, 2018)

[12] National Quality Forum Publications. Available at www.qualityforum.org/Publications/2017/08/Creating_a_Framework_to_Support_Measure_Development_for_Telehealth.aspx (last accessed 42, 2018)

[13] University of Arkansas Medical School South Central Telehealth Resource Center. Available at http://learntelehealth.org/category/connectedhealthresearch2017 (last accessed 42, 2018)

